# Higher enterococcus counts indicate a lower risk of colorectal adenomas: a prospective cohort study

**DOI:** 10.18632/oncotarget.25130

**Published:** 2018-04-20

**Authors:** Atsuko Kawano, Hideki Ishikawa, Michihiro Mutoh, Hiroyuki Kubota, Kazunori Matsuda, Hirokazu Tsuji, Kazumasa Matsumoto, Koji Nomoto, Ryuichiro Tanaka, Tomiyo Nakamura, Keiji Wakabayashi, Toshiyuki Sakai

**Affiliations:** ^1^ Institute of Gastroenterology, Zenjinkai Shimin-no-Mori Hospital, Miyazaki, Japan; ^2^ Department of Molecular-Targeting Cancer Prevention, Kyoto Prefectural University of Medicine, Kyoto, Japan; ^3^ Epidemiology and Prevention Group, Research Center for Cancer Prevention and Screening, National Cancer Center, Tokyo, Japan; ^4^ Yakult Central Institute, Tokyo, Japan; ^5^ Yakult Honsha European Research Center for Microbiology, ESV, Gent Zwijnaarde, Belgium; ^6^ Faculty of Nutrition, University of Kochi, Kochi, Japan; ^7^ Graduate Division of Nutritional and Environmental Sciences, University of Shizuoka, Shizuoka, Japan

**Keywords:** cancer prevention, colorectal cancer, enterococci, endoscopic polypectomy, intestinal microbiota

## Abstract

Intestinal bacteria play an important role in human health. This prospective cohort study aimed to investigate the relationship between the abundance of different intestinal bacteria and the risk of developing colorectal cancer (CRC). Fecal samples from CRC patients (*n =* 157) were collected at the start of the study wherein patients subsequently underwent endoscopy to remove polyps. Gut bacteria were isolated by using specific culture methods and the fecal counts of various bacteria were quantified by reverse-transcription-quantitative-PCR (RT-qPCR) assays. The obtained data were subjected to cohort analysis in relation to the incidence of colorectal adenomas after 4 years of intervention. No relationship was detected between the counts of major intestinal bacteria and the incidence of colorectal adenomas. However, interestingly, a significant negative correlation was noted between colorectal adenoma incidence and the counts of bacteria grown on Columbia blood agar base (COBA) (*P =* 0.007). The risk ratio of colorectal adenomas was 0.58 (95% CI: 0.35–0.96) in the group with the highest bacterial count compared to the lowest. Bacteria grown on COBA were more abundant in older patients, non-smoking patients, and patients with a lower body mass index. The RT-qPCR results revealed a significantly lower colorectal adenoma incidence in subjects with higher enterococcal count as compared to subjects with a lower count, with a risk ratio of 0.47 (95% CI: 0.30–0.76). Correlation of a higher enterococci count with a lower risk of CRC development suggests that certain Enterococcus strains may have adenoma suppressive effects.

## INTRODUCTION

The human gut is colonized by immense number and variety of bacteria that play an essential role in various aspects of human health and numerous disease [1–3]. Thus, it is reasonable to speculate that some intestinal bacteria might be associated with various diseases of the large intestine such as colorectal cancer (CRC), inflammatory bowel disease, irritable bowel syndrome, and coeliac disease [4–7].

Given that colorectal carcinogenesis is strongly influenced by environmental factors including lifestyle, the incidence of CRC varies greatly between geographical regions [8]. Epidemiological studies have shown that an excessive intake of lean meat, processed meat and alcohol are the risk factors for CRC. On the other hand, the intake of food rich in dietary fiber reduces risk of CRC [9]. Because these environmental factors may also affect the balance of intestinal bacteria, many researchers are interested in studying the role of intestinal microbiota in colorectal carcinogenesis and exploring whether gut microbes could be harnessed to develop useful preventive methods against CRC.

Lactic acid bacteria (LAB) have long been considered to have favorable effects on the large intestine in humans. Notably, several clinical studies have also reported a reduction in CRC risk following the administration of a suspension of live bacterial cultures (mainly LAB) to CRC patients [10, 11]. Thus, it is assumed that intestinal microbiota may play an important role in colorectal carcinogenesis; however, to date, no prospective clinical study has been conducted analyzing the intestinal microbiota of CRC patients and probing the correlation between gut bacteria and CRC development.

Herein, with an aim to address this deficiency, we carried out a prospective clinical study of 157 CRC patients to investigate the relationship between the types and abundance of different gut bacteria and the probability of CRC development. In a previous clinical study [10], we examined the intestinal flora of CRC patients at the initiation of the study (before endoscopy). Herein, we used this data to further evaluate the correlation of pre-intervention intestinal microbiota with the incidence of colorectal adenomas in the same patients at 4-years post-intervention follow-up.

## RESULTS

Of the 223 patients who submitted feces, 39 were excluded because they did not submit their feces on entry, and 12 were excluded because they did not undergo colonoscopy during the 4th year of intervention. Further, 15 were excluded because culture of their intestinal bacteria yielded a total bacterial count of less than 1 × 10^10^, indicating inappropriate culture. Accordingly, 157 patients were included in the analysis of cultures. Of note, 82 patients were from Group B (received dietary guidance and took a lactic acid product) and 75 were from Group D (received dietary guidance only). The baseline clinical characteristics of 157 patients are shown in Table 1. Regarding the baseline characteristics of Group B and D there are no significant differences between the two groups.

**Table 1:** Baseline characteristics at submission

	*n* = 157	
	Means ± SD	Range
Male sex, *n* (%)	133 (84.7)	
Age (years)	54.7 ± 6.2	41–65
Height (cm)	164.7 ± 7.2	144.0–181.5
Weight (kg)	64.3 ± 9.8	46.7–92.5
Alcohol drinker, *n* (%)	120 (76.4)	
Current smoker, *n* (%)	65 (41.4)	
Dietary intake		
Total energy (kcal/day)	2149 ± 392.2	1318.4–3854.7
Fat (g/day)	55.6 ± 13.8	19.2–99.5
Calcium (mg/day)	662.4 ± 243.5	228.3–1851.0
Carotene (mg/day)	2883.0 ± 1687.8	199.0–11139.4
Soluble dietary fiber (mg/day)	3.37 ± 1.16	0.96–8.30
Insoluble dietary fiber (mg/day)	11.85 ± 3.16	4.00–19.50
Total dietary fiber (mg/day)	15.22 ± 4.11	4.96–26.66

**Figure 1:**
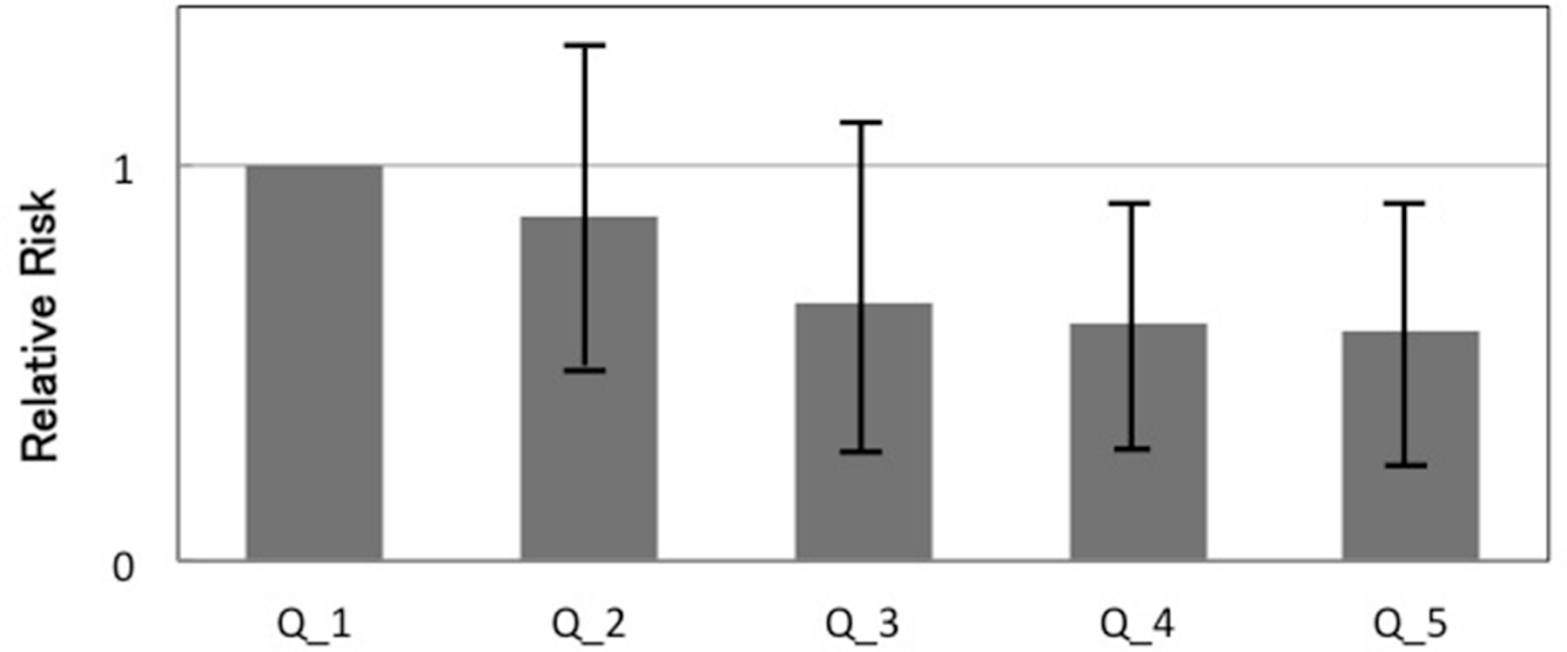
The incidence of colorectal adenomas according to the counts of the COBA-GPC Q1 represents the lowest counts of COBA-GPC, and Q5 is the highest. The relative risk of colorectal adenomas in Q1 is set as 1. COBA-GPC, Columbia blood agar - gram-positive cocci; Q1, 2, 3, 4, 5, Quintile1, 2, 3, 4, 5.

Table 2 shows the colony counts of intestinal microbiota and the incidence of colorectal adenomas in 157 patients from Groups B and D. We finally obtained 13 bacterial genera at this setting. The incidence of colorectal adenomas showed a significant decrease with an increase in the count of COBA-GPC among the 13 bacterial genera (Figure 1). In other genera, a trend was not found between the bacterial counts and the incidence of colorectal adenomas. The same analysis was performed separately in Groups B and D, but similar results were obtained; the size, number, grade and site of colorectal adenomas detected during the 4th year did not show any differences.

**Table 2:** Relationship between the colony counts of intestinal microbiota and the incidence of colorectal tumors

		Total	Q_1	Q_2	Q_3	Q_4	Q_5	*P* for trend
Total bacteria	mean	10.63	10.17	10.41	10.56	10.72	10.91	
	Positive/n		15/31	16/31	13/32	10/31	21/32	
	RR (95% CI)		1	1.06 (0.64–1.75)	0.83 (0.48–1.46)	0.66 (0.35–1.24)	1.35 (0.87–2.10)	0.74
*Bifidobacterium*	mean	10.27	9.6	9.93	10.18	10.35	10.63	
	Positive/n		15/32	10/31	20/32	14/30	16/32	
	RR (95% CI)		1	0.73 (0.39–1.37)	1.25 (0.78–1.99)	0.99 (0.58–1.69)	1.06 (0.64–1.76)	0.59
*Bacteroides*	mean	10.18	9.49	9.82	10.04	10.27	10.55	
	Positive/n		14/31	15/30	12/31	15/30	19/35	
	RR (95% CI)		1	1.10 (0.65–1.87)	0.85 (0.47–1.54)	1.10 (0.65–1.87)	1.07 (0.64–1.80)	0.72
*C. perfringens*	mean	6.82	1.24	1.36	2.83	4.4	7.49	
	Positive/n		10/30	16/33	19/32	15/30	15/32	
	RR (95% CI)		1	1.45 (0.78–2.69)	1.78 (0.99–3.18)	1.50 (0.80–2.78)	1.40 (0.75–2.63)	0.71
*Enterobacteriacae*	mean	8.19	5.61	6.43	6.95	7.61	8.84	
	Positive/n		15/30	16/33	15/32	14/30	15/32	
	RR (95% CI)		1	0.96 (0.58–1.60)	0.93 (0.56–1.56)	0.93 (0.55–1.57)	0.93 (0.56–1.56)	0.09
*Veillonella*	mean	7.80	2.25	4.07	5.54	6.91	8.53	
	Positive/n		16/31	18/33	18/31	10/31	13/31	
	RR (95% CI)		1	1.05 (0.66–1.67)	1.12 (0.71–1.77)	0.62 (0.33–1.15)	0.81 (0.47–1.38)	0.28
*Staphylococcus*	mean	3.94	2.24	2.35	2.8	3.51	4.59	
	Positive/n		12/29	13/32	18/32	17/31	15/33	
	RR (95% CI)		1	0.98 (0.53–1.79)	1.35 (0.80–2.30)	1.32 (0.77–2.27)	1.09 (0.61–1.94)	0.67
COBA-GPC	mean	8.64	6.16	6.71	7.49	8.2	9.24	
	Positive/n		19/29	16/28	12/28	15/38	13/34	
	RR (95% CI)		1	0.87 (0.57–1.32)	0.65 (0.39–1.08)	0.60 (0.37–0.96)	0.58 (0.35–0.96)	0.007
*Bacillus*	mean	7.14	2.26	2.68	3.56	4.97	7.83	
	Positive/n		17/31	17/31	15/32	11/30	15/33	
	RR (95% CI)		1	1.00 (0.63–1.57)	0.85 (0.52–1.39)	0.66 (0.37–1.18)	0.82 (0.50–1.35)	0.27
*Candida*	mean	5.07	2.23	2.31	2.58	3.52	5.75	
	Positive/n		14/30	16/32	15/32	15/31	15/32	
	RR (95% CI)		1	1.07 (0.63–1.79)	1.00 (0.59–1.70)	1.03 (0.61–1.75)	1.00 (0.59–1.70)	0.58
Lactobacilli	mean	7.99	4.19	5.27	6.21	7.21	8.74	
	Positive/n		16/30	19/33	12/34	12/31	16/29	
	RR (95% CI)		1	1.07 (0.69–1.68)	0.66 (0.37–1.16)	0.72 (0.41–1.26)	1.03 (0.64–1.65)	0.80

*C. perfringens, Clostridium perfringens*; COBA-GPC, Columbia blood agar - gram positive coccus.

RR, risk ratio; 95% CI: 95% confidential interval; Q_1.2.3.4.5, Quintile_1.2.3.4.5.

Positive number = The number of patients that observed at least one colorectal tumors in 4 years

The number of bacteria = log10/g wet feces.

Furthermore, the patients were classified into two groups by the count of COBA-GPC (by culture), and their baseline clinical characteristics were compared (Table 3). In patients with a low count of COBA-GPC, the mean age was significantly lower and the BMI was significantly higher. Smokers tended to have a lower COBA-GPC count, without a significant difference. No significant differences were noted in the intake of carotene, fat or dietary fiber between the two groups.

**Table 3:** Characteristics of two groups divided by the counts of cultured COBA-GPC

Mean (Range)	COBA-GPC (log10/g wet feces)		*P* value
	6.98 (4.37–7.63)	8.98 (7.67–10.14)	
	Means ± SD	Means ± SD	
Total number	85	72	
Age, years	54.6 ± 6.7	55.9 ± 5.4	0.02
Number of male^*^	74 (87.0)	59 (81.9)	0.21
Height (cm)	165.1 ± 7.3	164.3 ± 7.1	0.47
Weight (kg)	65.9 ± 7.3	62.4 ± 9.3	0.02
Body mass index (kg/m^2^ × 100)	24.0 ± 1.5	22.9 ± 1.5	0.01
Alcohol drinker^*^	69 (81.2)	51 (70.8)	0.26
Current smoker^*^	42 (49.4)	23 (31.9)	0.05
Dietary intake			
Total energy intake (kcal/day)	2149.0 ± 366.1	2150.4 ± 424.2	0.98
Fat intake (g/day)	55.5 ± 13.0	55.7 ± 14.9	0.96
Calcium intake (mg/day)	646.3 ± 210.9	682.0 ± 278.3	0.36
Carotene intake (mg/day)	2723.1 ± 1508.1	3076.6 ± 1875.6	0.19
Total dietary fiber (g/day)	14.9 ± 3.9	15.6 ± 4.3	0.29
Soluble dietary fiber (mg/day)	3.2 ± 0.9	3.4 ± 1.3	0.34
Insoluble dietary fiber (mg/day)	11.6 ± 3.1	12.1 ± 3.1	0.30

^*^Values are *N* (%).

In 138 samples, in which the total RNA was extracted, evaluable, quality data were obtained from 54 patients for the *L. lactis* subgroup, 120 for enterococci and 122 for streptococci. We assessed the risk of colorectal adenomas in patients with the three bacterial compositions of COBA-GPC (Table 4). The risk was lower in patients with a higher enterococcal count than those with a lower count. The difference was also significant after an adjustment for age, sex, BMI, smoking status, alcohol intake, and intervention groups. No association was found between the incidence of colorectal adenomas and the counts of lactococci or streptococci before or after adjustment.

**Table 4:** Risk of colorectal tumors and the counts of each bacteria using YIF-SCAN^®^ data

	counts of bacteria (log10/g)	Number of subjects	Number of events (%)	RR	95% CI	OR	95% CI
*L. lactis subgroup*	3.1–4.8	26	13 (50.0)	0.85	(0.48–1.52)	0.64	(0.19–2.18)
	5.0–7.4	28	12 (42.8)				
*Enterococcus*	3.1–6.1	70	44 (62.8)	0.47	(0.30–0.76)	0.23	(0.10–0.54)
	6.2–9.4	50	15 (30.0)				
*Streptococcus*	3.1–8.4	70	33 (47.1)	0.89	(0.59–1.34)	0.81	(0.38–1.70)
	8.5–10.3	52	22 (42.3)				

Abbreviations: L. lactis subgroup, Lactococcus lactis subgroup; RR, risk ratio; OR: odds ratio, adjusted by age, gender, BMI, smoking, alcohol intake and intervention groups.

95% CI, 95% confidence interval.

## DISCUSSION

In the present study, no relationship was found between the fecal counts of LAB, *Bifidobacterium* and *Bacteroides* and the incidence of colorectal adenomas after 4 years of intervention. However, a strong negative association was detected between the counts of enterococci cultured on COBA and the incidence of colorectal adenomas, suggesting that certain *Enterococcus* strains might prevent the development of colorectal adenomas.

It has been reported that both LAB and bifidobacteria are beneficial to humans, whereas *Bacteroides* include some harmful strains. However, we did not find any relationship of these bacteria with the incidence of colorectal adenomas. Instead, we found that enterococci, which have otherwise not been thoroughly investigated previously, are negatively associated with the incidence of colorectal adenomas.

Several epidemiological studies have reported that intestinal bacteria are involved in colorectal carcinogenesis. For instance, it has been reported that populations with a higher risk of CRC have more anaerobic bacteria, such as *Bacteroides* and *clostridia*, and fewer facultative anaerobic bacteria, such as streptococci, with a high ratio of anaerobes to facultative anaerobes [12, 13]. In contrast, other reports found no considerable differences in the intestinal microbiota or anaerobes-aerobes ratios between high-risk vs. low-risk CRC groups [14, 15]. In one report, patients with CRC were found to have a lower anaerobic-aerobic ratio [16]. One other study reported a higher ratio of anaerobes to aerobes in patients with adenomatous polyposis [17]. Two different Japanese studies have reported fewer bifidobacteria and more clostridia in patients with colorectal polyps or CRC [18], and an increased clostridia count in patients with recurrent sigmoid colon cancer [19]. On the other hand, other studies comparing the intestinal microbiota of patients with CRC or sporadic colorectal polyps with that of healthy adults reported no significant difference in the count of clostridia [20, 21]. As a result, it is difficult to formulate a general conclusion regarding the relationship between carcinogenesis and intestinal bacteria from the above-mentioned studies, mainly because these studies were descriptive epidemiological or cross-sectional studies, and not cohort studies that reported follow-up data of patients after the microbiota analysis. Moreover, these studies have methodological limitations, such as only using the data from cultures of intestinal bacteria.

In addition, the disparity in the results of the above-mentioned studies could be ascribed to the following reasons: (a) the configuration of the intestinal microbiota that could be involved in the carcinogenesis may have changed in patients with cancer or polyps or in postoperative patients because hemorrhage, stenosis, a surgical procedure or other factors may affect certain gut bacteria or the overall gut microbiota composition; (b) because culturing of intestinal bacteria requires very rigid anaerobic conditions, collected feces should be promptly put into an anaerobic bottle and transported to the laboratory under anaerobic conditions, but in the above-mentioned studies, the collection/transportation method might have differed among studies [22]; and (c) these studies were conducted in a statistically small number of subjects.

In the past two decades, the advent of molecular biological methods has enabled a rapid and specific detection of various bacterial species by using probes and primers targeting the 16S ribosomal RNA gene sequences. And this could be another reason for disparities in the results of ours study vs. other previous studies that used conventional culture-based methods [23]. Conventionally, the intestinal clostridia, with steroid nuclear dehydrogenase activity, have been thought to be involved in carcinogenesis. With the help of species-level molecular methods, *C. scindens, C. hiranonis* and *C. hylemonae* (in addition to some other species) have been identified as some of the major *Clostridium* species possessing a high steroid nuclear dehydrogenase activity [24]. However, despite the development of advanced molecular biological techniques, the relationship between CRC and the intestinal microbiota still remains unclear.

In the present study, we segregated the fecal bacteria by culturing methods and subsequently identified the bacteria by using specific RT-qPCR assays. As mentioned above, an inverse correlation was found between the fecal *Enterococcus* count and the incidence of colorectal adenomas. Enterococci exist as a normal member of the microbiota in the large intestine. Most of the *Enterococcus* species are believed to have no prominent effect on human health, and there have been no reports showing the possibility of enterococci preventing CRC development. Recently, we reported that heat-killed *Enterococcus faecalis* strain EC-12 weakly suppresses intestinal polyp development in *Apc*-mutant mice, Min mice, in part by attenuating β-catenin signaling and suppressing downstream molecules c-Myc and cyclin D1 [25]. Thus, it is speculated that this enterococci cultured on COBA may also inhibit adenoma growth through suppressing such growth-related molecules. However, *Enterococcus faecalis* (*E. faecalis*), among *Enterococcus* species, may also correlate with CRC development [26]. Moreover, this species produces free radicals, such as extracellular superoxide and hydrogen peroxide [27], that induce direct DNA damage in the large intestine, and hence this species might convert procarcinogens in food to carcinogens in the large intestine. However, we were not able to identify enterococci at the species-level and hence the role of specific enterococci in the prevention of development of colorectal adenomas remains unknown. Nevertheless, our results do underscore the need for further investigation to identify the *Enterococcus* species and/or strains involved in the prevention of colorectal adenomas as well as to elucidate the mechanisms underlying this association.

Our study had a few limitations. First, all study participants were former patients that underwent endoscopy for the removal of polyps or early stage adenocarcinomas. Therefore, the correlations observed in the current study might not be extrapolated to general population. The second limitation is the limited resolution due to the biased nature of bacterial analysis as only those bacteria that grew well within the particular culture assays and for which RT-qPCR primers were developed were assessed. Moreover, the possibility that many anaerobes must have remained uncultured or unidentified in the present study cannot be ruled out.

Nevertheless, our study did have several advantages as follows: (a) the incidence of colorectal adenomas was prospectively investigated after feces collection, (b) the number of subjects was much larger compared with previous studies, (c) patients with advanced CRC or those who had undergone resection of the large intestine were excluded, and (d) to reduce the chances of influence of the part of the feces sampled, the whole (not part) of the feces passed at one time was collected and cultured under the same conditions to prevent contamination by other bacteria. With these advantages, our study seems to indicate a reliable causal relationship between the intestinal microbiota and the incidence of adenomas in humans.

In summary, the findings from our prospective clinical study suggest that the composition of gut microbiota and the fecal counts of specific gut bacteria (in particular, *Enterococcus*) might be used as a prognostic factor to determine the risk of CRC re-emergence. In addition, the findings clearly call for further studies to explore if dietary interventions that can increase intestinal *Enterococcus* counts could also potentially decrease the chances of developing CRC in humans.

## MATERIALS AND METHODS

### Study design and subjects

Parts of the study design and methods of the present study were already reported in our previous study [10]. Briefly, the subjects were recruited at Osaka Medical Center for Cancer and Cardiovascular Diseases, which is located in a large city in Japan, between June 1993 and September 1997. The protocol of this study was approved by the Ethics Committee of this center, and all clinical investigations have been conducted according to the principles expressed in the Declaration of Helsinki. Written informed consent to participate in the study was obtained from all of the participants.

The candidates were men and women between 40 and 65 years of age, who had undergone total colonoscopy, who were found to have two or more adenomas or early CRC in the large intestine, and who had all of the lesions resected endoscopically within 3 months. Excluded from the study were patients with other malignant diseases, a history of familial adenomatous polyposis, serious disease, or resection of the stomach or intestine (excluding the appendix). Four hundred and seventy patients satisfied the inclusion criteria during the aforementioned period. All of these patients were asked to participate in this intervention study, and 410 (87%) agreed. Of these, 12 patients were excluded from the study because of the following noncompliance with the protocol: examination at entry revealed a bile duct cancer in one patient, gastric cancer in three patients, a history of gastrectomy in three patients, resection of the large intestine in one patient, and familial adenomatous polyposis in one patient. One patient was older than 66 years, another patient was younger than 40 years, and another had adenomas removed colonoscopically more than 3 months before. Accordingly, 398 patients were enrolled in the study.

The patients were randomly assigned to one of the four groups. Randomization was successfully performed and similar background characterization was obtained between the groups. Among them, Groups B and D are involved in the present study, i.e. Group B received dietary guidance and consumed a lactic acid product, and Group D received dietary guidance only. Other groups, Groups A and C are not used in the present study, i.e. Group A received dietary guidance and consumed wheat bran biscuit and Group C received dietary guidance and consumed wheat bran and a lactic acid product. The intervention lasted for four years.

The patients underwent total colonoscopy after 2 and 4 years of intervention to assess the recurrence of colorectal adenomas. The detected polyps were all removed and subjected to histological examination.

Feces were collected from 223 patients, who were in Groups B or D, to determine intestinal microbiota.

### Nutritional assessment and dietary guidance

All of the patients received a food-and-drink intake survey for three consecutive days. A well-trained dietitian interviewed each patient about his/her food-and-drink intake over approximately one hour, and the intakes of total energy, lipids, dietary fiber, vitamins, calcium, and other nutrients, including trace elements, were calculated.

Subsequently, all of the patients received dietary advice based on the data obtained so that the energy intake from fat would be within the range of 18 to 22% relative to the total energy intake to prevent the recurrence of colorectal adenomas.

### Collection and culture of feces

Feces were collected at least 2 weeks after the colonoscopy performed at study entry. Patients collected their feces on the day of, or the day before, the nutritional assessment before the initiation of intervention. They defecated in a plastic bag placed on a bowl, which was placed in the toilet bowl, to prevent the feces from being contaminated by other bacteria in the toilet bowl. The total amount of feces passed at one time was collected. The collected feces in the plastic bag were placed in another plastic bag, and then enclosed in an airtight bag together with an oxygen absorber, Ageless^®^ (Mitsubishi Gas Chemical Co., Inc., Tokyo, Japan) and a reagent tablet to detect oxygen. Then, the airtight bag was placed in a polystyrene foam case together with ice. Patients brought the cases to the hospital. At the hospital, the pH of the feces was immediately measured with a pH meter (F-8, Horiba Ltd., Kyoto, Japan).

Subsequently, an approximately 3 g aliquot of the feces was collected to prepare a sample, which was sealed in nitrogen gas and sent to the Yakult Central Institute while being kept in a cool place. At the Yakult Central Institute, quantitative culture was performed within 24 hours using selective media to allow for the growth of 13 bacterial genera, and the bacterial count of each genus per gram of wet feces was determined.

The remaining feces were diluted with the same amount of water as the weight of the feces and stirred well. The mixture was frozen at −30°C under vacuum and lyophilized. Then, the lyophilized feces were ground into a powder with a mill, frozen at −30°C, and used for molecular biological analysis.

### Quantification of fecal bacteria by RT-qPCR assays targeting bacterial 16S rRNA molecules

Among the 13 bacterial genera identified by culture, Columbia blood agar base (COBA), which is known to be selective mainly for enterococci, was used to culture bacteria belonging to three genera, *Enterococcus, Streptococcus*, and *Lactococcus*. We referred to these genera as COBA-gram-positive cocci (COBA-GPC).

To determine the bacterial composition of the COBA-GPC group, the lyophilized feces powder prepared as above was used. Each lyophilized fecal sample was mixed with a 30-fold volume of phosphate buffered saline (−) and the mixture was 10-fold diluted with phosphate buffered saline, assuming that the water content in lyophilized samples was 67%. A 200-mL aliquot of this suspension was mixed with 400 mL of RNA *later* (Thermo Fisher Scientific Inc.) in a 2.0-mL extraction tube. The mixture was centrifuged at 12,000 rpm for 5 min, after which the supernatant was removed by decantation and the pellet was stored at −80°C until RNA extraction. The total RNA from these stored samples was extracted using the method described previously [8]. The total RNA obtained was dissolved in 1.0 mL of nuclease-free water and the resulting solution was used as the total RNA stock solution. After excluding 19 samples because of poor storage condition or insufficient volume, 138 samples (72 from Group B and 66 from Group D) were subjected to total RNA extraction.

Fecal bacterial counts were quantified by employing a sensitive quantitative analytical system Yakult Intestinal Flora-SCAN^®^ (YIF-SCAN^®^) which is based on RT-qPCR assays targeting bacterial rRNA molecules [12]. The RNA extracted from the 138 samples were used as a template, and primer sets^9^ specific to three genera, enterococci, streptococci, and lactococci, were used to determine genus-specific counts. Briefly, RT-qPCR was performed with a Qiagen OneStep RT-PCR kit (Qiagen GmbH, Hilden, Germany). Each reaction mixture (10 μL) was composed of 1X Qiagen OneStep RT-PCR buffer, 0.5X Q-solution buffer, each deoxynucleoside triphosphate at a concentration of 400 μM, a 1:100,000 dilution of SYBR green I (BioWhittaker Molecular Applications, Rockland, ME), 0.4 μL of Qiagen OneStep RT-PCR enzyme mixture, and 5 μL of template RNA. Each primer set was added at a concentration of 0.6 μM. The reaction mixture was incubated at 50°C for 30 min for reverse transcription. The continuous amplification program consisted of one cycle at 95°C for 15 min, followed by 45 cycles at 94°C for 20 s, 60°C for 20 s, and 72°C for 50 s. Amplification and detection were performed in 384-well optical plates with an ABI PRISM 7900HT sequence detection system (Applied Biosystems, Foster, CA). Standard curves for the corresponding standard bacterial strains were generated by using Ct (threshold cycle) values of PCR reaction and the corresponding cell counts determined microscopically by DAPI (4ʹ,6-diamidino-2-phenylindole) staining method. To determine the target bacterial populations in the fecal samples, 1/20,000, 1/200,000, and 1/2,000,000 portions of the RNA solution were subjected to RT-qPCR. The Ct values in the linear range of the assay were applied to the analytical curve generated in the same experiment to obtain the corresponding bacterial count in each nucleic acid sample; this count was then converted to the count per sample. In our previous methodological studies, we validated that the counts enumerated by YIF-SCAN are equivalent to the bacterial counts obtained by culture and fluorescent *in-situ* hybridization methods and that the analytical sensitivity of YIF-SCAN is at least 100-fold higher than that of other DNA-based molecular methods [28–31]. Therefore, owing to its higher detection sensitivity, we opted to employ the RT-qPCR approach for the enumeration of COBA-GPC, because enterococci, streptococci and lactococci are present in subdominant levels in the human gut and hence might not be quantified precisely by routine DNA-based PCR methods [29].

The minimum detection sensitivity of these RT-qPCR assays was 3.0 log_10_ cells/g; and hence, bacterial counts of value 3.0 log_10_ cells/g or less were excluded from the data analysis.

### Statistical analysis

The patients were divided into five groups of approximately the same number according to the actual count of each of the 13 bacterial genera determined by the feces culture. The risk ratio for colorectal adenomas during the 4th year was calculated for each group relative to the group with the lowest bacterial count. Further, the trend between the bacterial count and the risk ratio was assessed based on the *P* for trend. Further analysis using the number, size and grade of the colorectal adenomas was also performed.

Subsequently, the patients were divided into two groups according to the COBA-GPC count, and baseline patient characteristics were compared. For comparison, the cut-off value was selected to maximize the sum of sensitivity and specificity to the incidence of colorectal adenomas using receiver operator characteristic curves. Differences in the percentages of males, current smokers and alcohol drinkers were assessed with the Mann-Whitney test, whereas differences in age, height, weight, body mass index (BMI), daily energy intake, and intakes of fat, calcium, carotene and dietary fiber were compared with the *t* test.

Furthermore, the patients with COBA-GPC were classified into two groups according to the bacterial count. Then, the risk ratio for colorectal adenomas during the 4th year was calculated relative to the group with the lower bacterial count (Reference 1). In addition, odds ratios were adjusted by logistic analysis, for age, sex, BMI, smoking status, alcohol intake and intervention groups. The level of significance was set at 0.05 in all of the analyses. IBM SPSS (ver. 20.0) was used for computer processing.

## Abbreviations

BMI: body mass index
COBA: Columbia blood agar base
CRC: colorectal cancer
GPC: gram-positive cocci
LAB: Lactic acid bacteria
RT-qPCR: reverse transcription-quantitative polymerase chain reaction
YIF-SCAN: Yakult Intestinal Flora Scan
